# Identification and characterization of water chestnut Soymovirus-1 (WCSV-1), a novel Soymovirus in water chestnuts (*Eleocharis dulcis*)

**DOI:** 10.1186/s12870-019-1761-7

**Published:** 2019-04-25

**Authors:** Fangpeng Zhang, Zuokun Yang, Ni Hong, Guoping Wang, Aiming Wang, Liping Wang

**Affiliations:** 1 State Key Laboratory of Agricultural Microbiology, Huazhong Agricultural University, Wuhan, Hubei People’s Republic of China; 2 Lab of Key Lab of Plant Pathology of Hubei Province, Huazhong Agricultural University, Wuhan, Hubei People’s Republic of China; 3 London Research and Development Centre, Agriculture and Agri-Food Canada, London, Ontario N5V 4T3 Canada

**Keywords:** *Caulimoviridae*, *Soymovirus*, Water chestnut, Virus-derived small RNA (vsRNA), Pararetrovirus, RNA sequencing

## Abstract

**Background:**

A disease of unknown etiology in water chestnut plants (*Eleocharis dulcis*) was reported in China between 2012 and 2014. High throughput sequencing of small RNA (sRNA) combined with bioinformatics, and molecular identification based on PCR detection with virus-specific primers and DNA sequencing is a desirable approach to identify an unknown infectious agent. In this study, we employed this approach to identify viral sequences in water chestnut plants and to explore the molecular interaction of the identified viral pathogen and its natural plant host.

**Results:**

Based on high throughput sequencing of virus-derived small RNAs (vsRNA), we identified the sequence a new-to-science double-strand DNA virus isolated from water chestnut cv. ‘Tuanfeng’ samples, a widely grown cultivar in Hubei province, China, and analyzed its genomic organization. The complete genomic sequence is 7535 base-pairs in length, and shares 42–52% nucleotide sequence identity with viruses in the *Caulimoviridae* family. The virus contains nine predicated open reading frames (ORFs) encoding nine hypothetical proteins, with conserved domains characteristic of caulimoviruses. Phylogenetic analyses at the nucleotide and amino acid levels indicated that the virus belongs to the genus *Soymovirus*. The virus is tentatively named *Water chestnut soymovirus-1* (WCSV-1). Phylogenetic analysis of the putative viral polymerase protein suggested that WCSV-1 is distinct to other well established species in the *Soymovirus* genus. This conclusion was supported by phylogenetic analyses of the amino acid sequences encoded by ORFs I, IV, VI, or VII. The sRNA bioinformatics showed that the majority of the vsRNAs are 22-nt in length with a preference for U at the 5′-terminal nucleotide. The vsRNAs are unevenly distributed over both strands of the entire WCSV-1 circular genome, and are clustered into small defined regions. In addition, we detected WCSV-1 in asymptomatic and symptomatic water chestnut samples collected from different regions of China by using PCR. RNA-seq assays further confirmed the presence of WCSV-1-derived viral RNA in infected plants.

**Conclusions:**

This is the first discovery of a dsDNA virus in the genus *Soymovirus* infecting water chestnuts. Data presented also add new information towards a better understanding of the co-evolutionary mechanisms between the virus and its natural plant host.

**Electronic supplementary material:**

The online version of this article (10.1186/s12870-019-1761-7) contains supplementary material, which is available to authorized users.

## Background

Members of the *Caulimoviridae* family are plant pararetroviruses that contain a double-strand DNA (dsDNA) genome and replicate through an RNA intermediate. The genomes of caulimoviruses are singular, circular dsDNA with two to three discontinuous positions, approximately 7.2–9.3 kb in size, and have one open reading frame (ORF) as shown in *Petunia vein clearing virus* (PVCV) of the *Petuvirus* genus to eight ORFs such as in the members of the genus *Soymovirus.* The *Caulimoviridae* family has eight definitive genera: *Badnavirus, Caulimovirus, Cavemovirus, Petuvirus, Solendovirus, Tungrovirus, Soymovirus* and *Florendovirus*, in which there are currently 68 species [1–3]. The *Soymovirus* genus includes *Soybean chlorotic mottle virus* (SbCMV), *Blueberry red ringspot virus* (BRRV), *Peanut chlorotic streak virus* (PCSV), *Cestrum yellow leaf curling virus* (CmYLCV) and unassigned *Cranberry red ringspot virus* [2, 4–7]. The genomes of soymoviruses are approximately 8 kb in length and contain seven to eight ORFs, and a large intergenic space region between ORF VI and VII that serves as a promoter [6, 7].

Water chestnut (*Eleocharis dulcis*) is a seasonal aquatic vegetable that is cultivated worldwide. The plant is economically valuable in the food industry due to its popularity and the high nutritional value of its edible bulb. As the plant is usually clonally propagated via vegetative bulbs, viral agents can easily be transmitted to next generation and be spread worldwide by germplasm exchange. Such transmission of viral agents may cause significant economic losses as bulb quality and yield are often reduced in virally infected plants. From 2012 to 2014, water chestnut plants in the fields in Fanggaoping Town, Tuanfeng County, Hubei province, China, showed chlorosis and streaking symptoms. The exclusive viral disease in China known so far to infect water chestnut is *Cucumber mosaic virus* [8]. Thus, the etiology of the viral disease in water chestnut plants remains unclear.

High throughput sequencing of nucleic acids within the host provides an unbiased approach to identify unknown pathogens. The combination of high throughput sequencing for small RNAs (sRNA) and RNA sequencing with bioinformatics analysis and validation by PCR amplification is an approach that has potential to identify and characterize plant viruses and explore host-virus interactions [9–16]. Here, we used this approach and identified a new-to-science virus from water chestnut plants. We further determined its genomic organization and sequence characteristics. The vsiRNA profiles and RNA transcriptional activity based on high throughput sequencing were further analyzed and evaluated.

## Results

### Identification of a novel soymovirus (tentatively named WCSV-1) in water chestnut samples by deep sequencing and subsequent bioinformatics analyses

**Fig. 1:**
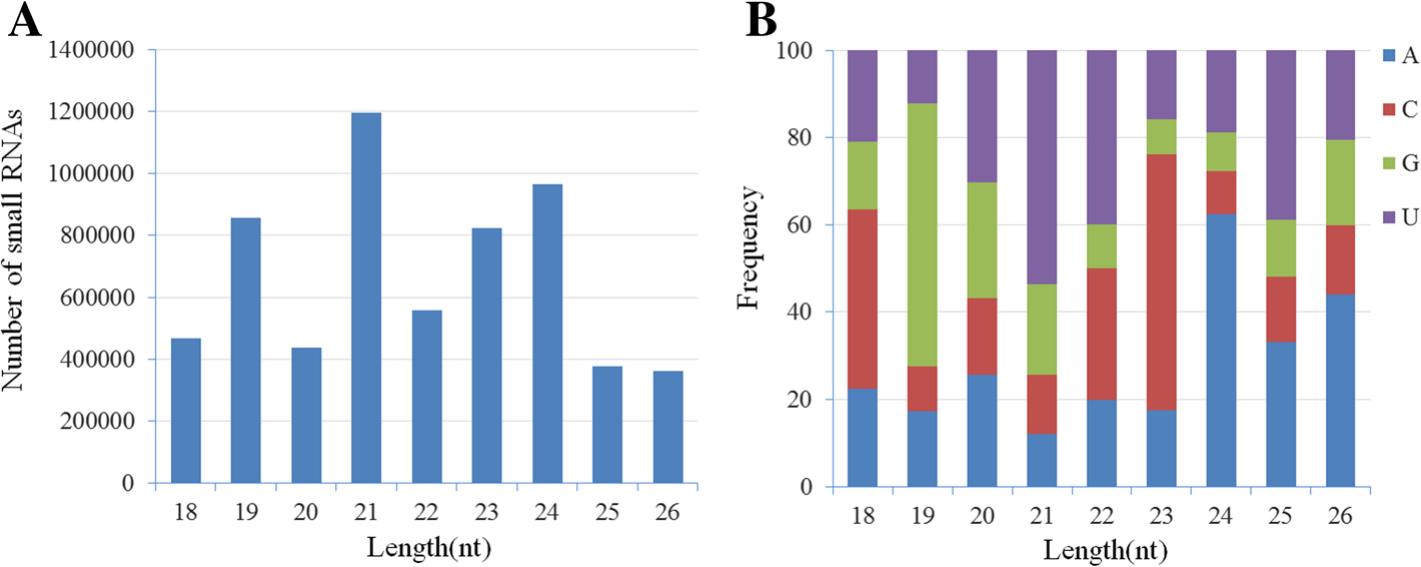
Illumina sequencing results. A Graph showing the size distribution of the small RNAs sequence from water chestnut samples with WCSV-1. B The relative frequency of each nucleotide A (blue), C (red), G (green), and U (purple) in the 5′ terminal nucleotide position in sRNA molecules 18–26 nt in length-only high quality reads shown

To identify the unknown virus associated with disease in water chestnut plants, a deep sequencing approach was used to identify sRNA sequences including those that may be produced by a virus in samples from symptomatic water chestnut plants. A total of 21,089,253 raw reads were obtained by Hiseq high throughput Solexa sequencing from sRNA isolated from fresh leaf samples. The sequence reads were filtered by the removal of those that were low quality (containing 3′ and 5′ adaptor contaminants) and < 18 nt or > 26 nt in size, and the remaining 6,041,373 clean reads were kept for subsequent analyzes. The majority of these clean sRNA reads were 21 nt in length (*n* = 1,195,408; 19.8%), followed by 24 nt (*n* = 966,046; 16%), 19 nt (*n* = 856,162; 14.2%), and 23 nt (*n* = 823,614; 13.6%; Fig. 1A). There was a clear bias in the 5′-terminal nucleotide depending on the size of the sRNA. The most common 5′ nucleotide in 20–22 nt sRNAs was U, which occurred in 54% of 21 nt, 40% of 22 nt, and 30% of 20 nt sRNAs. For the other sRNAs, the 5′ terminal nucleotide was A in 63% of the 24 nt sRNAs, G in 60% of the 19 nt sRNAs, and C in 59% of the 23 nt sRNAs (Fig. 1B).

**Fig. 2:**
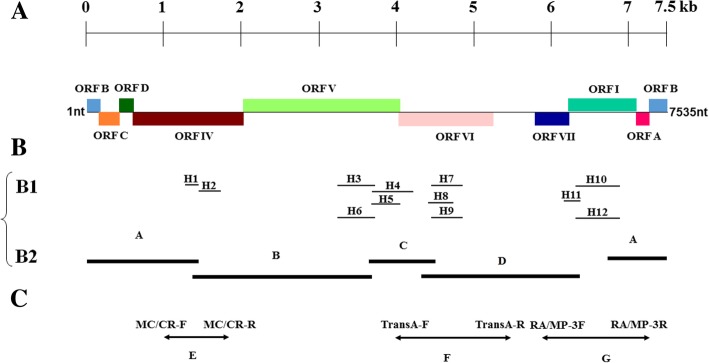
The cloning strategy for the WCSV-1 full-length genome. **a** A linear genomic map of WCSV-1 is shown, in which the putative ORFs are represented by rectangles; **b** the 12 contigs (H1-H12) obtained from small RNA sequencing are presented as gray lines (B1), and the four fragments (labeled A-D) amplified by the first PCR reaction using primers based on the contigs are indicated by black lines (B2); **c** three fragments **e**–**g** amplified by the second PCR using primers from the obtained clones to fill in the gaps are shown as double arrows. The relative positions of primers are also indicated

Based on the sequencing data, 12 contiguous sequences were identified (H1-H12) that were 200 to 345 nt in length. The amino acid sequences of the predicted ORFs coded by H1-H12 were close matches to sequences found in the *Soymovirus* genus in the family *Caulimoviridae* (Fig. 2). The newly virus identified was tentatively designated water chestnut soymovirus virus-1 (WCSV-1).

### Determination and analyses of the genomic sequence of WCSV-1

To further characterize the genome and taxonomic status of WCSV-1 within the *Caulimoviridae* family, the viral dsDNA genome was cloned and sequenced. Seven primer sets were designed based on the homology of the WCSV-1 sequence to PCSV sequences to amplify specific viral sequences (Additional file 1: Table S1). PCR products covering the full genome were obtained. The approximate positions of the contiguous sequences and seven cloned fragments (A-G) from PCR products covering the whole WCSV-1 genome are indicated in Fig. 2. The full-length genomic sequence of WCSV-1 was 7535 bp (GenBank Accession No. KU365408). The AT composition in WCSV-1 genome was 66%.

The genomic sequence of WCSV-1 had 42–52% nucleotide identity to those of members of the *Caulimoviridae* family, and shared the highest similarity with members of the *Soymovirus* genus. WCSV-1 had the highest sequence similarity to SbCMV (49%), PCSV (52%), CmYLCV (48%), and BRRV (50–51%). The predicted amino acid sequence of WCSV-1 had the highest homology with that of PCSV in the *Soymovirus* genus across the proteins encoded by ORF I, ORF IV, ORF V, ORF VI, and ORF VII (with a similarity ranging from 38 to 69%). In addition, the most conserved regions of polyfunctional protein (pol) encoded by ORF V in WCSV-1 were closely related to other caulimoviruses at the amino acid level (with a similarity ranging 44–68%) and at nucleotide acid sequence level (with an identify ranging 41–60%) (Table 1).

**Table 1:** Pairwise sequence identity and similarity alignments for water chestnut soymovirus-1 (WCSV-1) ORFs and other *Caulimoviridae* family members

Genus	Virus	Genome size (bp)	Nucleotide identity(%)		Amino acid identity(similarity)(%)				
			Full genome	ORF V	ORF I	ORF IV	ORF V	ORF VI	ORFVII
*Badnavirus*	BSV-GF	7263	42	26			20(31)		
	ComYMV	7489	45	26			21(33)		
	CSSV	7161	43	27			20(33)		
*Caulimovirus*	CaMV-XinJiang	8060	44	49	27(47)	27(47)	35(54)	16(28)	7(17)
	CERV	7932	44	49	23(44)	24(42)	33(52)	18(28)	*
	DMV-Portland	7916	45	41	28(47)	22(35)	29(44)	17(33)	7(16)
	FMV	7743	43	50	28(49)	25(44)	33(51)	19(33)	5(12)
*Cavemovirus*	CsVMV	8159	46	52			19(34)		
*Petuvirus*	PVCV	7206	44	20			23(33)		
*Solendovirus*	SPVCV-Dom1	8837	46	49			21(34)		
	TVCV	7767	48	49			21(35)		
*Soymovirus*	BRRV-BRRSV24	8265	50	59	34(53)	28(47)	46(66)	21(35)	25(44)
	BRRV-Coville546	8299	50	57	31(48)	29(47)	44(64)	21(34)	25(44)
	BRRV-Darrow5	8302	51	58	31(49)	29(46)	46(65)	20(34)	25(44)
	BRRV-NJ	8303	50	60	32(51)	27(45)	46(66)	20(34)	23(39)
	CmYLCV	8253	48	53	29(52)	27(46)	44(62)	17(31)	*
	PCSV	8174	52	55	48(69)	32(51)	50(68)	19(38)	31(51)
	SbCMV	8178	49	55	31(54)	31(49)	48(65)	18(33)	29(49)
*Tungrovirus*	RTBV-PH	8002	46	27			19(31)		

Percentage similarities are listed in Parentheses. The following abbreviations are used to indicate virus names: *BSV-GF* Banana streak virus-GF, *ComYMV* commelina yellow mottle virus, *CSSV* Cacao swollen shoot virus, *CaMV-XinJiang* cauliflower mosaic virus-XinJiang, *CERV* Carnation etched ring virus, *DMV-Portland* Dahlia mosaic virus-Portland; *FMV* Figwort mosaic virus, *CsVMV* Cassava vein mosaic virus, *TVCV* Tobacco vein clearing virus, *PVCV* Petunia vein clearing virus, *SPVCV-Dom1* Sweet potato vein clearing virus-Dom1, *BRRV* Blueberry red ringspot virus, *CmYLCV* Cestrum yellow leaf curling virus, *PCSV* Peanut chlorotic streak virus, *SbCMV* Soybean chlorotic mottle virus, *RTBV-PH* Rice tungro bacilliform virus-PH.“*” indicates no ORF VII in CERV and CmYLCV

**Fig. 3:**
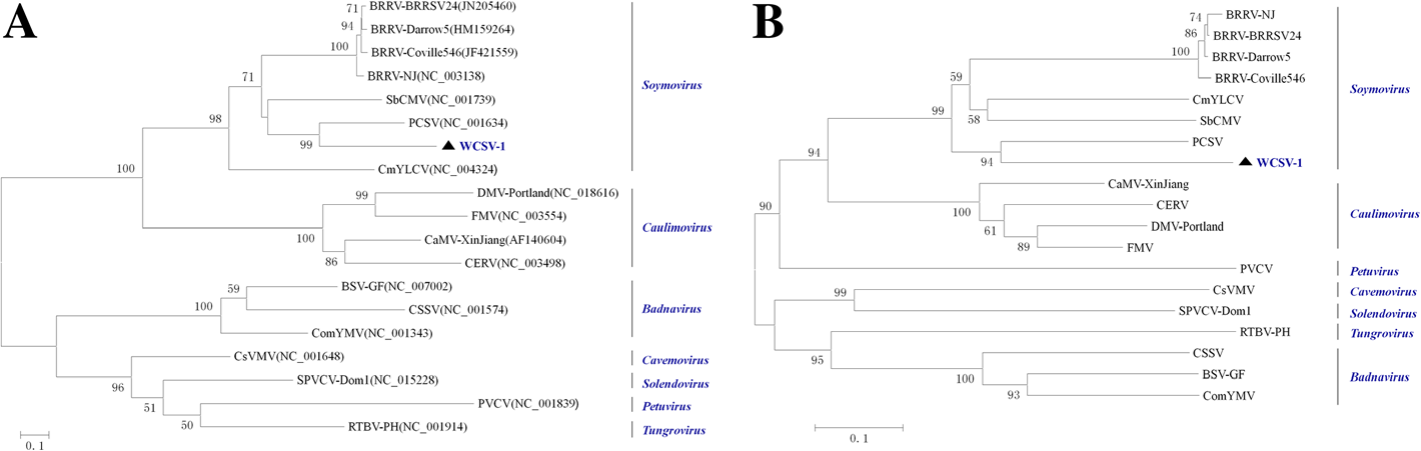
Phylogenetic tree showing the relationships between WCSV-1 and selected numbers of the *Caulimoviridae* family. **a** A phylogenetic tree was constructed based on full-length genomic sequences for WCSV-1 and other *Caulimoviridae* specified as virus names (abbreviation) followed by sequence accession numbers. **b** Phylogenetic tree based on the amino acid sequences encoded by ORF V. The phylogenetic trees were estimated using the neighbor-joining method with 1000 bootstrap replicates. Bootstrap values > 50% are shown at branch numbers. The bar represents 0.1 substitutions per site. The following abbreviations are used to indicate virus names in the phylogenetic tree analysis. BRRV, Blueberry red ringspot virus; SbCMV, Soybean chlorotic mottle virus; PCSV, Peanut chlorotic streak virus; CmYLCV, Cestrum yellow leaf curling virus; DMV-Portland, Dahlia mosaic virus-Portland; FMV, Figwort mosaic virus; CaMV-XinJiang, cauliflower mosaic virus-XinJiang; CERV, Carnation etched ring virus; BSV-GF, Banana streak virus-GF; CSSV, Cacao swollen shoot virus; ComYMV, commelina yellow mottle virus; CsVMV, Cassava vein mosaic virus; SPVCV-Dom1, Sweet potato vein clearing virus-Dom1; PVCV, Petunia vein clearing virus; RTBV-PH, Rice tungro bacilliform virus-PH. The position of WCSV-1 is indicated by the triangle

**Fig. 4:**
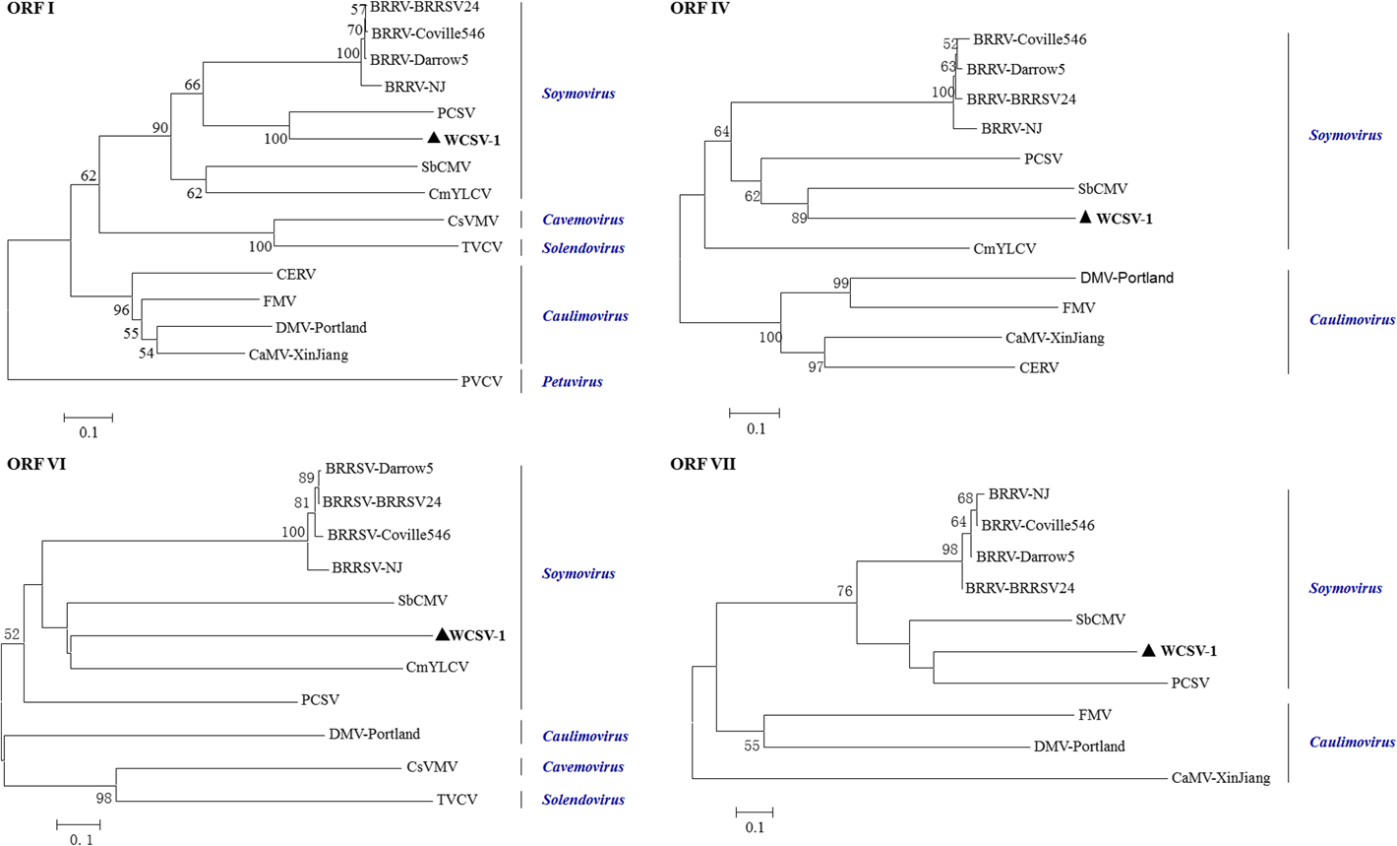
Phylogenetic tree analysis of WCSV-1 compared with selected numbers of *Caulimoviridae* family based on ORF I, IV, VI and VII. Phylogenetic trees were constructed based on the amino acid sequence encoded by each of the ORFs as described in Fig. 3 and using the same abbreviations. The position of WCSV-1 is indicated by the triangle

Phylogenetic relationships between WCSV-1 and randomly selected members of the *Caulimoviridae* family were analyzed based on their full genomic sequences (Fig. 3a) and putative polymerase protein encoded by ORF V (Fig. 3b). Both phylogenetic trees showed similar topology, indicating that WCSV-1 clustered with members of the *Soymovirus* genus, and formed a separate branch close to PCSV. This pattern identified WCSV-1 as a new member of the *Soymovirus* genus that was distinct to other well characterized species. Similarly, the deduced ORF I, IV, VI, and VII sequences also placed WCSV-1 in the *Soymovirus* genus with SbCMV, PCSV, CmYLCV, and BRRV (Fig. 4). Taken together the results indicated that the WCSV-1-Hubei isolate analyzed in the present study is a new species of the *Soymovirus* genus in the *Caulimoviridae* family.

### Characterization of the genomic organization of WCSV-1

**Fig. 5:**
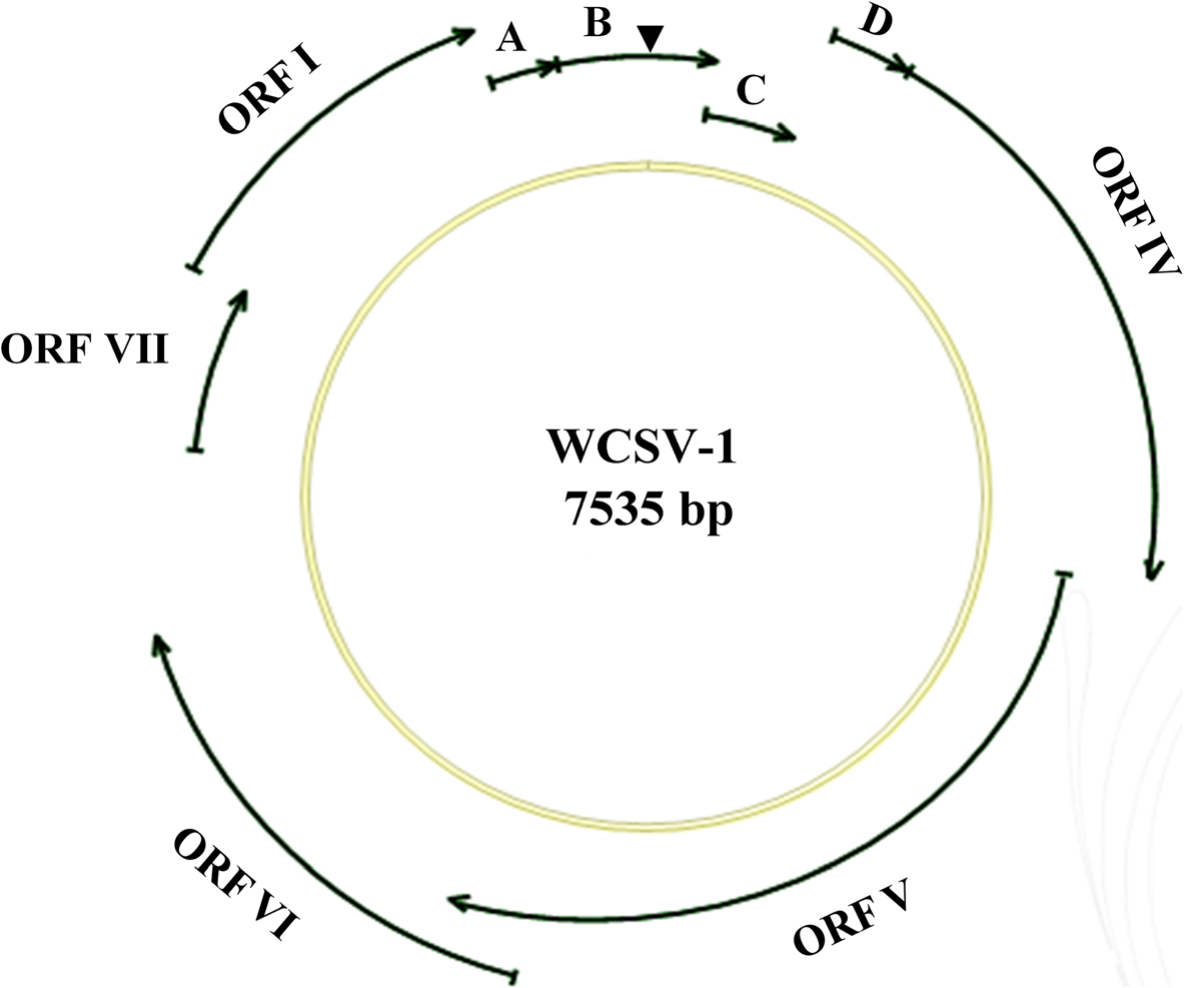
WCSV-1 genomic organization based on the cloned viral DNA sequences. The circle represents the dsDNA genome. The outer arrows indicate the position of nine ORFs, designated as ORF I, IV, V, VI, VII and A, B, C, D, respectively. “▼” indicates the starting position of the 12 nt primer binding site

To better understand the novel WCSV-1 virus, we annotated the viral genome using bioinformatics approach. A graphical representation of the WCSV-1 genomic organization including predicted ORFs and their approximate locations on the viral dsDNA is presented with the annotated sequence (Figs. 2a and 5). The organization of the WCSV-1 genome is similar to most of other *Soymovirus* members. The WCSV-1 ORFs share significant homology with their counterparts in previously described *Soymovirus* species (Table 1). Nine putative ORFs encoding proteins were predicted on the positive strand of the WCSV-1 genome using the ORF Finder program available at the NCBI website (http://www.ncbi.nlm.nih.gov/) (Figs. 2a and 5). In known *Soymoviruses*, ORF I encodes a cell to cell movement protein. ORF IV encodes the viral coat protein (CP), containing the CX_2_CX_4_HX_4_C sequence referred as the zinc-finger protein. ORF V encodes the viral replicase that has three conserved domains; the aspartic proteinase (AP), reverse transcriptase (RT), and ribonuclease H (RNase H). ORF VI encodes a protein termed the inclusion body or the translational transactivator protein [17]. ORF VII encodes a hypothetical protein necessary for PCSV infectivity [5]. There are three small hypothetical proteins encoded by other ORFs that have undefined functions [18]. Each ORF in WCSV-1 was annotated and is presented below.

**Fig. 6:**
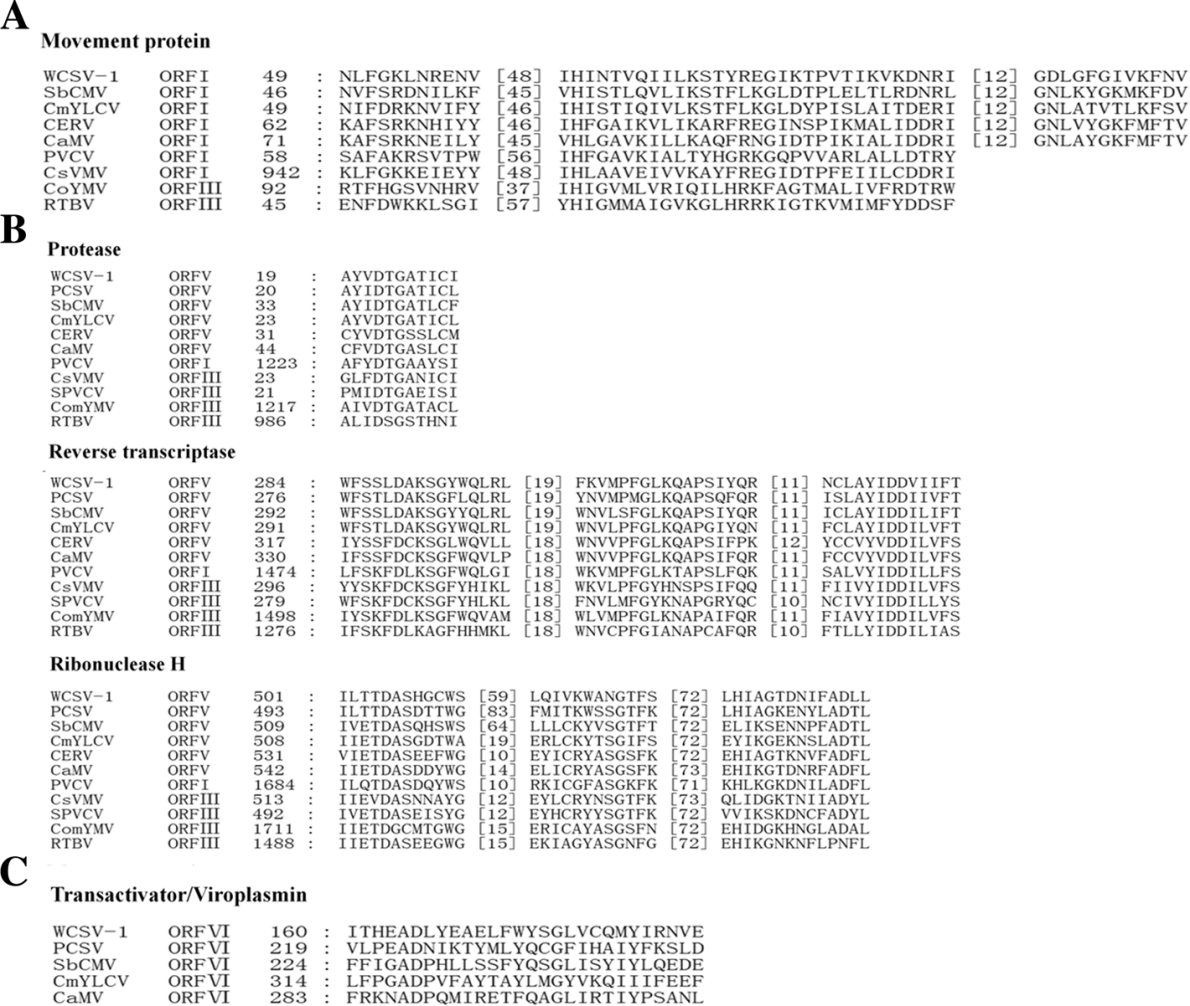
Alignments of highly conserved motifs encoded by WCSV-1 ORF V (**a**), ORF I (**b**), and ORF VI (**c**) with the corresponding regions from other viruses in the *Caulimoviridae* family. The abbreviated name of the virus, ORF designation, and the amino acid position starting from the N terminal (numerical) are indicated for each sequence in the left hand columns. The spacing between amino acid sequences is shown in square bracket. The abbreviations are defined in Fig. 3

ORF I (6,225–7118 nt) encodes a putative cell to cell transport movement protein (MP) that is estimated to contain 297 amino acids and has an estimated 33.8 kDa molecular weight, which is slightly smaller than the corresponding protein from known soymoviruses. It shares 44–69% amino acid similarity with the corresponding proteins from other *Caulimoviruses* and *Soymovirus*, particularly in the conserved RNA-binding domain. The deduced amino acid sequence in WCSV-1 also contains a putative transport domain with the sequence GDLGFGIVKFNV (amino acid positions 151–162), and the sequence DNR (135–137 aa), a conserved DXR motif (Fig. 6a).

ORF A (7,115–7294 nt) encodes a 59 amino acid protein that has a predicated molecular weight of 6.8 kDa. ORF B (7,298–173 nt) encodes a protein that is estimated to contain 136 amino acids and be approximately 16.1 kDa in size. The ORF B gene includes a 12 primer binding site (5′-TGGTATCAGAGC-3′) located in positions 1–12 nt and 22–33 nt, which is complementary to the consensus tRNA^Met^, and is predicated to be a minus-strand primer binding site that is highly conserved in all members of the *Caulimoviridae* family [6, 19]. ORF C (166–441 nt) and ORF D (438–629 nt) encode proteins that are estimated to be 10.8 kDa (91 amino acid) and 6.8 kDa (63 amino acid), respectively. The proteins encoded by ORFs A, B, C, and D do not share any similarities with the corresponding proteins encoded by other soymoviruses.

ORF IV (636–2072 nt) encodes the viral CP and is estimated to contain 478 amino acids and have a molecular weight of 56.8 kDa. This is one of the more conserved genes among caulimoviruses [4]. It contains a zinc binding motif composed of the sequence CX_2_CX_4_HX_4_C (X represents any amino acid), which is a conserved motif of an RNA-binding domain. In WCSV-1 the precise sequence is: CKCWLCQKEGHYANEC (amino acid position 405–420). In addition, the WCSV-1 ORF IV contains a K rich (37% K) core upstream of the zinc binding motif (amino acid positions 380–431) [4].

ORF V (2,065–4107 nt) encodes the RNA-dependent DNA polymerase (RT) that contains 680 amino acids and is estimated to be 78 kDa. The RT contains some of the most conserved amino acid motifs indicating a common evolutionary origin of *Caulimoviridae* family [1, 20] As expected, the RT from WCSV-1 contains the conserved amino acid sequence (YIDDVIIF) for the putative RT domain found in other caulimoviruses at amino acid positions 351–358 [4]. The consensus sequences for RNase H were also identified (Fig. 6b). In addition, the AYVDTGATIC sequence at amino acid positions 19–28 in WCSV-1 is similar to a putative AP active site motif AX2DXGXT reported in other caulimoviruses [17, 21]. Thus, the putative protein encoded by ORF V includes highly conserved motifs of AP, RT and RNase H (Fig. 6b).

ORF VI (4080–5309 nt) encodes a hypothetical protein that is 409 amino acids and estimated to be 47 kDa, which corresponds to the putative transactivator protein in caulimoviruses [18]. WCSV-1 was most closely related to SbCMV, PCSV, and BRRV isolates, and the similarity was particularly notable for the left half of the full-length sequence encoded by ORF VI (amino acid positions 146–351 in WCSV-1 vs. 205–407 in PCSV; and 145–353 in WCSV-1 vs. 211–426 in SbCMV). ORF VI in WCSV-1 also contains another well-conserved region that is common to the *Soymovirus* genus at positions 176–182 (GLVCQMY; Fig. 6c) [4, 6]. Phylogenetic trees showed WCSV-1 clustered in a separate clade, near CmYLCV and SbCMV providing further support that WCSV-1 is a *Soymovirus* (Fig. 4).

ORF VII (5780–6232 nt) encodes a hypothetical protein with 150 aa and an estimated molecular weight of 17 kDa. The protein had some similarity (23–31%) to the amino acid sequences of the corresponding protein in other *Soymovirus* isolates, and was slightly similar (5–7%) to the *Caulimovirus* genus (Table 1)*.* As with the other ORFs, the phylogenetic analysis indicated WCSV-1 was most closely related to PCSV (Fig. 4).

Non-coding regions approximately 470 bp in length were found in the viral DNA genome. The genome contains a potential TATA box (TATATAA) located at position 5477–5483 nt and a polyadenylation signal (AATAAA) at position 5743–5748 nt.

### Characteristics of virus-derived small RNAs (vsRNAs) from WCSV-1

**Fig. 7:**
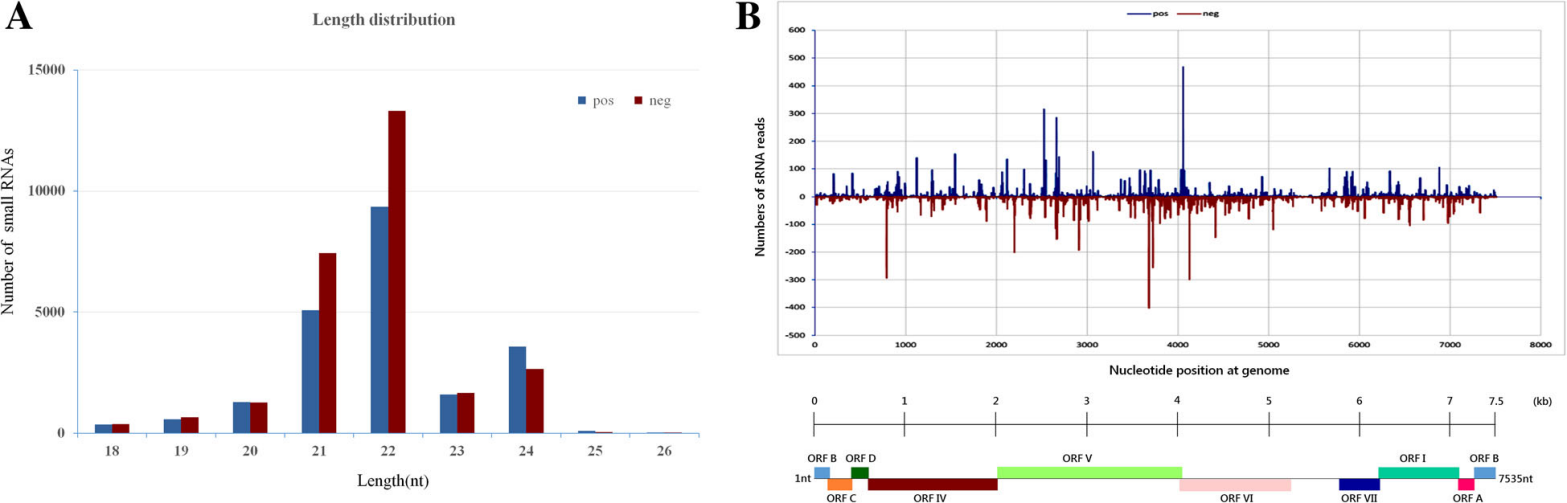
Characteristics of the WCSV-1 vsRNA pool from water chestnut samples. **a** The length of the WCSV-1 vsRNA was determined by sequencing and the number of vsRNAs of each length is shown. The vsRNA pool is divided into the positive (blue) and negative (red) strands. **b** Genome-wide map of WCSV-1 vsRNA at the single-nucleotide resolution. A linear genomic map of WCSV-1 is shown. The positive strand is shown in blue and the negative strand is shown in red

**Fig. 8:**
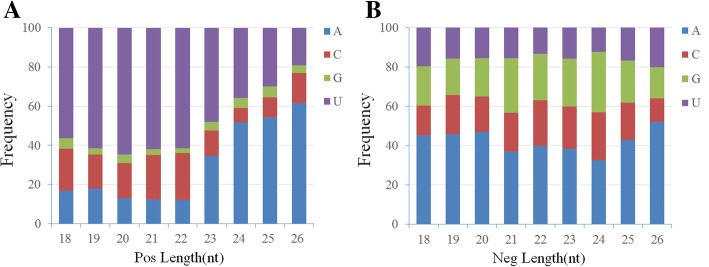
The relative frequency of 5′ terminal nucleotides in water chestnut vsRNA pool. The relative frequency of each nucleotide A (blue), C (red), G (green), and U (purple) in the 5′ position on the positive strand (**a**) and negative strand (**b**) of WCSV-1 vsRNAs is indicated and sorted by length of the vsRNA

Based on the described genomic features of WCSV-1, the characteristics of the vsRNAs were analyzed in terms of size abundance, positive and negative strand use, and distribution along the WCSV-1 genome (Fig. 7). The vsRNA profiles indicated that the entire WCSV-1 genome was mapped by 49,400 reads (0.82% of total reads). A slightly greater number of vsRNAs derived from negative strand (27,454 reads) than from the positive strand (21,946 reads) based on aligning the vsRNAs to the WCSV-1 sequences. The most abundant WCSV-1 vsRNAs were 22 nt (positive strand: 13,304; negative strand: 9352) or 21 nt (7437 positive strand; negative strand 5074). The vsRNA from the positive and negative strands were discontinuous and covered the WCSV-1 genome unevenly. Three hotspots were observed; one on the positive strand between positions 4057–4077 the most conserved vsRNA sequence was 5′-UUUGCCGAUCUAUUAACUAGA-3′; and two on the negative strand at positions 3681–3702 and 3724-3745, the most conserved vsRNA sequences were 5′-CCAAUCAGUACAUUCCCAGGUA-3′ and 5′-ACCGUCUUCAGUACAAAACAGG-3′, respectively. There were also four positions within the RT gene at positions 2524-2545, 2661-2681, 3724-3745, and 2197–2218 nt that were associated with a large number of vsRNA reads. Finally, there were a large number of vsRNAs from the negative strand of ORF VI positions 789–810 (5′-GAACCAAGUAGUGAUGAUUCAG-3′) and the viral CP gene positions 4127–4147 (5′-GGACAUCCACAGCCAGAUAGA-3′). The 5′ terminal nucleotide was determined for each of the vsRNAs in size. There was a strong preference for U (62/61%) > C (22/24%) > A (12.4/12.2%) > G (2.8/2.4%) in the 5′ terminal position in positive strand vsRNA that were 21–22 nt in length. The negative strand vsRNA (21–22 nt) showed a preference for A (37/40%) > G (28/23%) > C (19/23%) > U (15/13%) in the 5′ terminal nucleotide position (Fig. 8).

### Detection of WCSV-1 in water chestnut plants by PCR

To determine the prevalence of WCSV-1 infection in diseased water chestnut plants PCR was used to detect the presence of WCSV-1 in plant samples. Sixty-four (64) samples representing 24 cultivars of water chestnut collected from the Hubei and Guangxi provinces were 100% positive for WCSV-1. The three different primer sets used to amplify WCSV-1 sequences resulted in differences in ability to generate the target amplicon (Table 2). For example, all 32 cladode clonal propagation samples from Tuanfeng County stored at the greenhouse of the National Indoor Conservation Center for Virus-free Germplasm in Fruit Crops, generated the expected 543 bp amplicon using the primer set CP-F/R but only 18 of 32 (56%) of these same samples were positive using the primer set of MP-F (5′-GAATATCAAGAGGAATCAGG-3′)/MP-R (5′- CTAGTGTAGATTCTGTCCAG-3′) to amplify a 650 bp fragment of the MP region. Thus, the WCSV-1 genome was detectable in all symptomatic and asymptomatic water chestnut plant samples.

**Table 2:** Summary of the WCSV-1 PCR-positive results for water chestnut samples from Hubei and Guangxi Province

Time of collection	Location of collection	Sample tissues of detection	Cultivars	Sample of detection (number)	Positive PCR (numbers)			Positive rate (%)
					MP-F/R	CP-F/R	RA-F/R	
2014/4/13	Tuanfeng County	Cladode	Tuanfeng	32	18	32	–	100
2014/5/18	Tuanfeng County	Cladode^※^	Tuanfeng	20	20	20	–	100
2014/11/25	Tuanfeng County	Bulbs	Tuanfeng	10	–	8	10	100
2014/11/26	Tuanfeng County	Roots	Tuanfeng	10	–	10	10	100
2014/6/5	Wuhan Vegetable Science Research Institute	Cladode	SanJiang	1	–	1	–	100
			TaWan	1	–	1	–	
			GuiGang	1	–	1	–	
			YangLiu	1	–	1	–	
			JianLi No.1	1	–	1	–	
			YangDian	1	–	1	–	
			GuiLin No.1	1	–	1	–	
			ZhangLe	1	–	1	–	
			GuiTi No.2	1	–	1	–	
			1106	1	–	1	–	
			ZhanJiang	1	–	1	–	
			LiPu	1	–	1	–	
			YiChang	1	–	1	–	
			ChangDeWuLing	1	–	1	–	
2014/11/26	Guangxi Academy of Agricultural Sciences	Cladode	Wild water chestunt	1	–	0	1	100
			FangLin	1	–	1	1	
			HengXian	1	–	1	1	
			GuiLin	1	–	1	1	
			GuiTi No.1	2	–	2	2	
			GuiTi No.2	2	–	2	2	
			LiPu No.1	1	–	1	1	
			LiPu No.2	1	–	1	1	
2014/12/24	Jinxiu County	Bulbs	GuiTi No.2	8	–	8	8	100

Note: “--”indicates no detection; “Cladode^※^” indicates Cladode samples from tissue culture shoots

The virus distribution in the plant was also assessed by determining whether virus was present in samples from the corms, roots, and cladode. Ten corm and 10 root samples were randomly selected from a pool of 32 samples maintained at the greenhouse of the National Indoor Conservation Center for Virus-free Germplasm in Fruit Crops, for testing. All (100%) of the samples were positive for WCSV-1 by PCR with the primer set of RA-F/R that targets the partial RT gene (Primer provided in Additional file 1: Table S1). 80% (8/10) and 100% (10/10) samples of corm and root were PCR positive for WCSV-1 using the primer set CP-F/R that targets the CP gene. Finally, 20 samples of tissue culture shoots were randomly selected from a pool of 32 samples and 100% of the samples were PCR positive for WCSV-1 infection using either the CP-F/R or MP-F/R primer pairs (Table 2).

A survey was conducted utilizing 14 randomly selected samples of water chestnut, from the germplasm stores at the Wuhan Vegetable Science Research Institute, and 10 cladode and 8 bulb samples from the germplasm of Guangxi Academy of Agricultural Sciences and Jinxiu county of Guangxi province, respectively. All (100%) of the surveyed samples from both locations were PCR positive for WCSV-1 using the RA/F-R primer set (Table 2; Additional file 2: Figure S2). The sequence similarity for the partial *cp* gene (543 bp) and the RA gene (875, 879, and 881 bp) ranged from 97 to 100% and 83–100%, respectively in eight of the isolates from the Guangxi and Hubei provinces. Sequencing of randomly selected PCR products generated with the three sets primers confirmed the PCR positive results. Given that all of the plants sampled were PCR positive for WCSV-1, it raised the possibility that the virus exists as an endogenous pararetrovirus (EPRV) sequence in the water chestnut. Sequencing the water chestnut genome in combination with further additional experiment is necessary to validate the hypothesis.

### Identification of WCSV-1-derived viral RNA in water chestnut by Illumina RNA-seq analysis

**Fig. 9:**
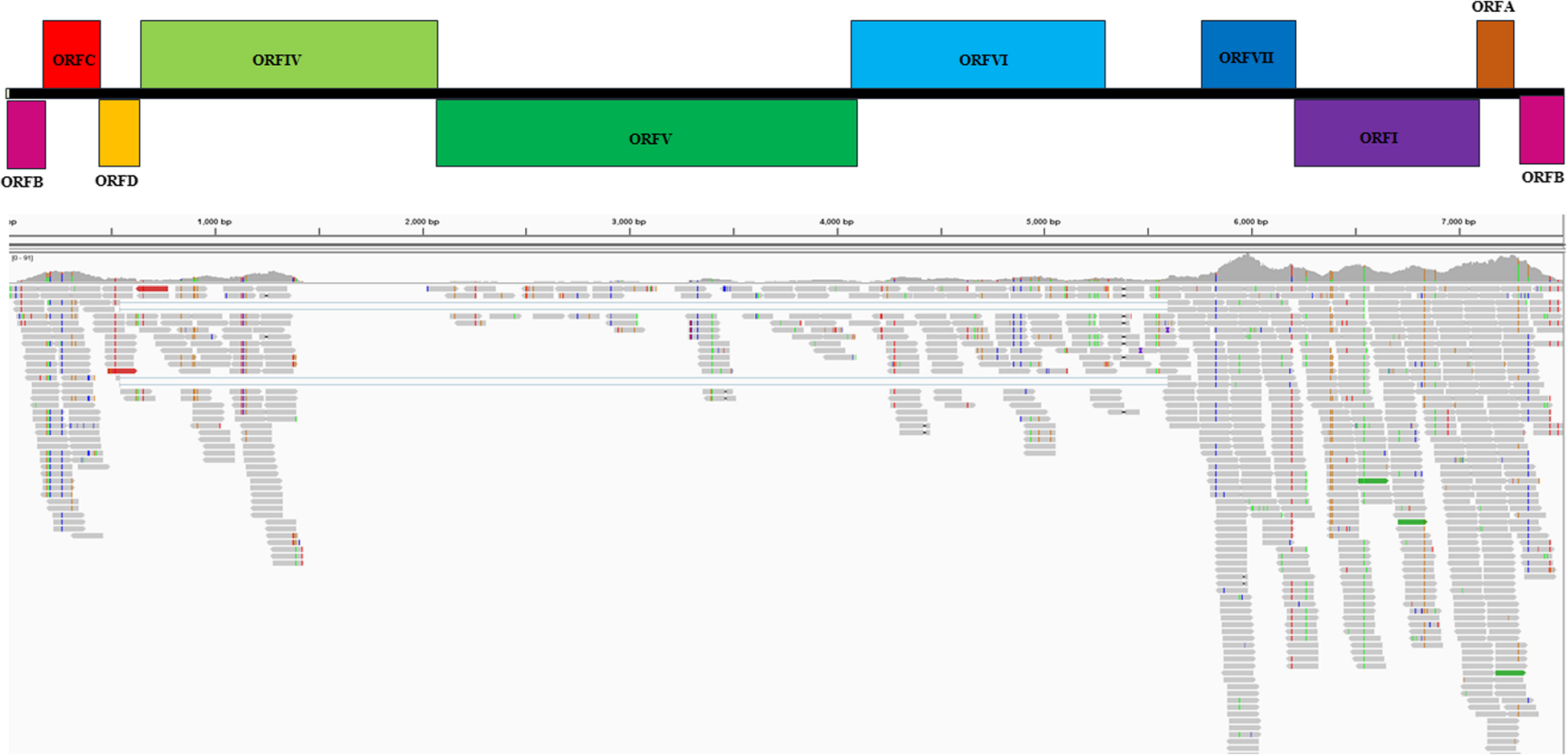
Overview of water chestnut transcriptome reads distribution along the WCSV-1 genomic sequence. The color is shown according to the overlapping of genomics coordinates between the predicted ORFs and read counts

In order to confirm the viral expression in water chestnut plants and explore virus-plant interactions, we conducted RNA-seq assays in leaf tissues of virally infected plants. We identified a total of 1665 reads that matched the viral genomic sequence including 107 mapped to ORF C, 30 to ORF D, 175 to ORF IV (CP), 102 to ORF V (RT), 175 to ORF VI (a hypothetical protein), 322 to ORF VII (a hypothetical protein), 489 to ORF I (MP), 171 to ORF A, 94 to ORF B (Fig. 9). Apparently the virus in water chestnut sample had low expression levels.

## Discussion

WCSV-1 was discovered through a high throughput sequencing-based survey of water chestnut viruses collected from Tuanfeng county, Hubei province, China. Here, the genomic characteristics and sequences of WCSV-1 were determined (Table 1; Figs. 3, 4, 5 and 7b). Bioinformatics analysis of sequences from WCSV-1 indicated the presence of conserved viral features shared with the MP, CP, AP, RT, and RNaseH proteins in *Caulimovirida*e viruses (Fig. 6), suggesting that WCSV-1 is a novel member of *Caulimovirida*e family distinct from other well characterized viruses in the genus *Soymovirus* (Figs. 3 and 4) [3]. WCSV-1 had a smaller genome (7535 bp) but more ORFs (nine) predicated by computer analysis, in comparison with other soymoviruses that have average 8.1–8.3 kb genomes and seven to eight ORFs [1]. There were also more ORFs (ORFs A-D) between ORF I and ORF IV, where there are normally two (*Caulimovirus*) or three (*Soymovirus)* ORFs. The putative ORFs A-D (< 10 kDa for two proteins encoded by ORF A and ORF D) in WCSV-1 had no significant homology with the analogous proteins in other caulimoviruses. The tRNA^Met^ primer binding site within ORF D as distinct from ORF Ib in SbCMV and ORF A in PCSV. In addition, a TATA box (TATATAA) located at position 5477–5483 nt, and a downstream polyadenylation signal (AATAAA) at 5743–5748 nt were predicated in WCSV-1, which require further study. Overall our findings support that WCSV-1 is a novel member belonging to the *Soymovirus* genus of the *Caulimoviridae* family.

It was reported that members in *Caulimoviridae* are able to integrate into the host genomes; for example: Banana streak virus a *Badnavirus* that infects *Musa* spp. and *Dioscorea* spp., Fig badnavirus-1 (FBV-1) that infects *Ficus* spp. [22–25]*,* and Tobacco vein clearing virus a *Cavemovirus* that infects *Nicotiana* spp. [26]; The infectivity of EPRVs depends on the host plant; in some cases EPRV is non-infectious [27–30], while in others such as EPRVs in bananas, tobacco, rice, and petunias are infectious [26, 31–33]. Moreover, previous studies have suggested that corm tip tissue culture, mechanical inoculation, inter-specific hybrids, and environmental factors may trigger EPRV escape from the host genome to cause infection [27, 34]. Soymoviruses have been reported as episomal virus infections affecting blueberry, peanuts, cestrum, and soybean plants [4–7, 35–37]. We attempted to address the question of whether WCSV-1 is an integrant and exist as an episomal form or cause active infection, and whether WCSV-1 could produce the disease seen in the cultivate water chestnut plants.

In this study, we found that (1) all of the water chestnut shoots were PCR positive for WCSV-1 irrespective of whether they were collected in cultivated fields (symptomatic and asymptomatic plants) or as germplasm repositories (Table 2); (2) WCSV-1 sequences were detected throughout the plants in different tissue including cladode, roots, bulb, and meristem tissue culture shoots from the propagation of protocorm-like bodies (Table 2); In addition, the observed WCSV-1 transcriptional activity is an additional signal of the viral presence and activity (Fig. 9), which is associated with viral titer and phenotype [15]. In summary, our findings led us to raise the question that WCSV-1 is an episomal virus or/and EPRV from the *Soymovirus* genus. As reported, southern blotting analysis, situ hybridization and transmission experiments need to be identify if WCSV-1 integrated into the genome of water chestnut host. If WCSV-1 is an EPRV, the integrant may in fact be protective against related *Soymovirus* infection through gene silencing mechanisms [30, 37]. However, as noted EPRVs can reactivate under the right circumstances [31, 33, 38, 39]. Deep sequencing of the vsRNA pool in WCSV-1 infected water chestnut plants could provide valuable leads for understanding how the host plant responds to WCSV-1 by gene silencing (Figs. 7 and 8). For example, the WCSV-1 vsRNA were predominately 22 nt in size, suggesting that the water chestnut homologues of Dicer ribonucleases 4 (DCL4) and DCL2 were working synergistically to make the vsRNAs, similar to previous findings in *Arabidopsis* plants [40, 41]. Interestingly, the most prevalent 5′-terminal nucleotide at the 21–24 vsRNA is “A” and “U” in the negative and positive polarity of WCSV-1, respectively (Fig. 8). It is well known that 21-nt vsRNAs are preferentially loaded into AGO1 and AGO4 [42] and targeting specific regions of the dsDNA virus sequence is an antiviral defense mechanism [18]. The WCSV-1 vsRNA composition and patterns such as the 5′-terminal nucleotide bias may reflect the specific interaction between WCSV-1 and its host water chestnut. Therefore, the sRNA characteristics from WCSV-1 in the water chestnut may provide clues about the mechanism of plant host defense for soymoviruses by gene silencing homogenous sequences [43–45].

## Conclusions

To our knowledge, this is the first report of a DNA virus in the genus *Soymovirus* infecting with water chestnuts, a previously undescribed *Soymovirus* species infecting with water chestnut in China. High throughput small RNAs sequencing and RNA sequencing, viral sequence specific PCR amplification in combination with bioinformatics were used to identify a circular dsDNA virus from water chestnut plants, and determined its genomic organization and sequence characteristics. These obtained results of sRNA characteristics, RNA transcriptional activity in combination with high frequency positive for WCSV-1 sequence in the water chestnut, provide new insights into a novel model system of virus-host co-evolution mechanism. Further research will be explored, in particular the relationship between biological analysis of WCSV-1 as well as investigation of water chestnut plants infecting with WCSV-1 in the field.

## Methods

### Plant material

In January 2013, 14 water chestnut bulbs (‘Tuanfeng’, a widely grown cultivar) were collected from a commercial field in Tuanfeng County, Wuhan city, Hubei province in China. The bulbs were separated into 32 separate potted plants maintained in the experimental greenhouse at the National Indoor Conservation Center for Virus-free Germplasm in Fruit Crops, Huazhong Agricultural University. We monitored viral diseases by randomly sampling the corms, roots, and tissue culture shoots randomly from the plants for PCR analyses with virus-specific primers.

In June of 2014, 14 water chestnut cladode samples showing either chlorosis, dwarfing, leaf distortion, or no obvious symptoms were collected from the germplasm of the Wuhan Vegetable Science Research Institute, Hubei province for PCR analyses. In November and December of 2014, we randomly collected and conducted PCR assays of 10 samples of cladode tissue and eight samples of corm from an experimental field of the Guangxi Academy of Agricultural Sciences, and a commercial field in Jinxiu County, Guangxi province, respectively.

### Small RNA sequencing and bioinformatics analysis

For sRNA sequencing, two microgram of total RNAs were extracted from the young leaves of six greenhouses grown, potted, water chestnut ‘Tuanfeng’ cv samples stored at the greenhouse of the National Indoor Conservation Center for Virus-free Germplasm in Fruit Crops, using the Trizol reagent (Invitrogen, USA). In brief, low molecular weight sRNA was enriched and isolated by polyacrylamide gel electrophoresis. The sRNA molecules (< 30 nt) were ligated to a 3′ and 5′ adaptor. Reverse transcription-PCR, gel electrophoresis, and nucleic acid precipitation were performed to construct the sRNA library, and then sequenced using the Illumina HiSeqTM 2000 platform by BioMarker Technologies Company (Beijing, China). Raw Illumina sRNA reads were filtered and trimmed by removing the 5′ and 3′ primer contaminants, insert tags, polyA tags, and fragments shorter than 18 nt and longer than 26 nt to result in clean reads. Clean sRNAs were assembled into contiguous sequences using Velvet with a 17 k-mer value [46]. The contiguous sequences were aligned with known virus genomes from the National Center for Biotechnology Information (NCBI, USA) database using BLASTN and BLASTX to identify similar sequences present in the water chestnut samples. In addition, the virus-derived small RNA (vsRNA) profile and the viral sense and antisense genomes were determined using the Bowtie software allowing up to two mismatches [47].

### PCR amplification and molecular cloning of water chestnut Soymovirus-1 (WCSV-1) genomic sequence

PCR was performed using total DNA isolated from water chestnut ‘Tuanfeng’ cv sample as template using *Taq* DNA polymerase and LA polymerase (Takara Company, Dalian, China). Four sets of primers (Additional file 1: Table S1) were designed using Oligo7 based on contiguous sequences identified using sRNA sequencing that corresponded to homologous viral sequences [48]. Sequence gaps were filled using PCR with three sets of virus-specific primers designed from the obtained sequences (primer sequences are shown in Additional file 1: Table S1). The PCR reactions consisted of the following: 50 ng DNA, 10 mM dNTPs, 1 U *Taq* polymerase, 10 mM of each primer, and sterile water to a final volume of 25 μL. The amplified products were separated by electrophoresis using a 1.2% agarose gel. The PCR products were isolated with the QIAquick PCR purification Kit and cloned into the pMD18-T vector (Takara Company), and then transformed into *E.coli* DH5α cells. At least three independent clones from each PCR product cloned into the pMD18-T vector were submitted and performed for sequencing by Jinsirui Biotechnology and Service Co. Ltd. (Nanjing, Jiangsu province, China).

### WCSV-1 genomic sequence analysis

The amplicon sequences were assembled using the program ContigExpress (Vector NTI Advance 11.5) with a > 99% similarity threshold for each overlapping region to obtain the complete genomic sequence of WCSV-1. Conserved protein domains were identified using the conserved domain database. Sequence similarity searches were performed using the online tools BLASTN and BLASTX from the NCBI. Pairwise alignments of sequence identity and similarity at the amino acid level were performed using the Needleman-Wunsch Global Alignment in the European Molecular Biology Open Software Suite [49]. Phylogenetic analyses based on multiple sequences alignments at the nucleotide and amino acid levels were performed using the programs Clustal X 1.83, and MEGA6 [50, 51]. Alignments of highly conserved motifs encoded by WCSV-1 ORF V, ORF I, and ORF IV with the corresponding regions from other viruses in the *Caulimoviridae* family were performed using the program Gendoc [52].

### Extraction of total genomic DNA from water chestnut

Total DNA was extracted from the cladode, corm, and root of symptomatic and asymptomatic water chestnut seedlings, and the leaves of taro plants (as a negative control) using the cetyltrimethyl ammonium bromide (CTAB) method with minor modifications. Briefly, plant tissues (about 0.1 g) that had high fiber content were snapping frozen with liquid nitrogen and then ground into a fine powder. The powder was transferred into 1 mL of 2% CTAB solution containing of 2% polyvinylpyrrolidone (PVP), 100 mM Tris-HCl (pH 8.0), 1.4 M NaCl, 20 mM EDTA, and 0.2% 2-mecaptoethanol. Total DNA was extracted from the solution using previously described methods [53]. Finally, the total DNA was desiccated and then dissolved in 100 μL of deionized sterile distilled water for immediate use or storage at − 80 °C. The quality of the template DNA was evaluated by agrose gel electrophoresis prior to use in PCR analyses.

### RNA sequencing and data analysis

Total RNA was isolated from water chestnut samples collection from Guangxi province. RNA quality and amounts were determined using Nanodrop (Thermo Scientific, CA, USA), Qubit 2.0 (Life Technologies, CA, USA), Aglient 2100 (Agilent Technologies, CA, USA). The rRNAs were removed using the Epicentre Ribo-ZeroTM kit. mRNA was purified, and cut into small fragments. The first cDNA was synthesized using random hexamer primer and Reverse Transcriptase. Second strand cDNA synthesis was subsequently performed using DNA Polymerase I and RNase H. The sequencing adaptors were ligated to cDNA after adding A tailing. PCR products were purified with AMPure XP beads regent (Beckman Coulter, Beverly, USA) to enrich the cDNA library. Each library had an insert size of 200–300 bp sequenced in paired-end reads of 150 bp using the Illumina HiSeq X-ten platform by BioMarker Technologies Company (Beijing, China). Raw reads were filtered and trimmed via the removal of containing a high content (> 5%) of unknown bases (N), and adaptor-polluted low-quality reads using the internal software. The obtained clean reads were used to analyze expression levels of ORFs from WCSV-1. Bowtie2 followed by the software of IGV (Integrative Genomics Viewer) were employed to align reads against WCSV-1 genome and further to observe the overview of identified reads distribution along the whole viral genome sequence [54, 55].

## Additional files

Additional file 1: **Table S1.** Primer sequences used for PCR amplification of the full genomic sequence of WCSV-1. (DOCX 20 kb)

Additional file 2: **Figure S2.** 1.2% agarose gel electrophoresis of PCR products of WCSV-1 infecting water chestnut samples of “Tuanfeng” cultivar. A1: Direct PCR amplification of 650 bp products of WCSV-1 ORF I from 12 cladode samples of water chestnut using the primers of MP-F/R. M: Marker II (TIANGEN Biotech, Beijing Co., Ltd.), Line 1–12: Cladode tissue samples; ck+: Small RNA sequencing water chestnut sample as positive control; ck1-:ddH_2_O, ck2-: Taro sample. B1 and B2: Direct PCR amplification of 543 and 875 bp products of WCSV-1 ORF IV and ORF VI from 10 bulb tissue samples of water chestnut using the primers of CP-F/R and RA-F/R, respectively. M: Marker II (TIANGEN Biotech, Beijing Co., Ltd.), Line 1–10: bulb samples; ck+: Small RNA sequencing water chestnut sample as positive control; ck2-:Taro sample. C1 and C2: Direct PCR amplification of 543 and 875 bp products of WCSV-1 ORF IV and ORF VI from 10 root tissue samples of water chestnut using the primers of CP-F/R and RA-F/R, respectively. M: Marker II (TIANGEN Biotech, Beijing Co., Ltd.), Line 1–10: root samples; ck+: Small RNA sequencing water chestnut sample as positive control; ck2-: Taro sample. The samples were maintained at the greenhouse of the National Indoor Conservation Center for Virus-free Germplasm in Fruit Crops. (DOCX 330 kb)

## Abbreviations

AP: Aspartic proteinase
BRRV: *Blueberry red ringspot virus*
CmYLCV: *Cestrum yellow leaf curling virus*
CTAB: Cetyltrimethyl ammonium bromide
dsDNA: Double-strand DNA
EPRV: Endogenous pararetrovirus
ORF: Open reading frame
PCSV-K1: *Peanut chlorotic streak virus*-K1
PVP: Polyvinylpyrrolidone
RT: Reverse transcriptase
SbCMV-JA: *Soybean chlorotic mottle virus*-JA
vsRNA: Virus-derived small RNAs
WCSV-1: Water Chestnut Soymovirus-1
