# Supplementary material for: Identification and characterization of water chestnut Soymovirus-1 (WCSV-1), a novel Soymovirus in water chestnuts (Eleocharis dulcis)
Source: BMC Plant Biol. 2019 Apr 25;19:159. doi: 10.1186/s12870-019-1761-7 (PMC6482551; doi:10.1186/s12870-019-1761-7)
Supplement: Supplementary file 1 — Table S1. Primer sequences used for PCR amplification of the full genomic sequence of WCSV-1. (DOCX 20 kb) [file 12870_2019_1761_MOESM1_ESM.docx]

**Additional file 1: Table S1.** **Primer sequences used for PCR amplification of the full genomic sequence of WCSV-1.**

| Primer | Sequence (5'→3') | Location on genome (bp) | Name of amplified fragment | Size of fragments (bp) |
| --- | --- | --- | --- | --- |
| MC-F | GGAGTACAAGTTCATTTCGAAC | 6762-6783 | A | 2231 |
| MC-R | CATTGTTTGTTTTTCTGATTG | 1437-1457 |  |  |
| CR-F | TGTGACCCTTGTTATTTAGAGA | 1374-1395 | B | 2340 |
| CR-R | TCTTCCAGCTTTACCTGGGAAT | 3692-3713 |  |  |
| RA-F | GGATGCTCTTATGAGCCAATCA | 3666-3687 | C | 875 |
| RA-R | GTTCTTGGACTTCCTGCATGAT | 4519-4540 |  |  |
| AM-F | AAAAAGGATCTTGGCCCAGT | 4377-4396 | D | 2061 |
| AM-R | AACTCCTTGTTCTCCCTGCAT | 6417-6437 |  |  |
| MC/CR-F | AAGGGAAGGACAGTTGCAAA | 998-1017 | E | 712 |
| MC/CR-R | GAAATGTTTTTTCCTATGTGG | 1689-1709 |  |  |
| TransA-F | CTGATAACATCTTTGCCGAT | 4046-4065 | F | 1428 |
| TransA-R | ATCTAAAACCTATACAGCGTT | 5453-5473 |  |  |
| RA/MP-3F | AACAGCAAAACACCCGGTAA | 5929-5948 | G | 1435 |
| RA/MP-3R | ACCATTCTCAATTGGTTCCTG | 7343-7363 |  |  |
