# Supplementary material for: Identification and characterization of water chestnut Soymovirus-1 (WCSV-1), a novel Soymovirus in water chestnuts (Eleocharis dulcis)
Source: BMC Plant Biol. 2019 Apr 25;19:159. doi: 10.1186/s12870-019-1761-7 (PMC6482551; doi:10.1186/s12870-019-1761-7)

**Additional file 2: Figure S2**. **1.2% agarose gel electrophoresis of PCR products of WCSV-1 infecting water chestnut samples of “Tuanfeng” cultivar.**

**A1**: Direct PCR amplification of 650 bp products of WCSV-1 ORF I from 12 cladode samples of water chestnut using the primers of MP-F/R. M: Marker II (TIANGEN Biotech, Beijing Co., Ltd.), Line 1-12: Cladode tissue samples; ck+: Small RNA sequencing water chestnut sample as positive control; ck1-:ddH_2_O, ck2-: Taro sample. **B1** and **B2**: Direct PCR amplification of 543 and 875 bp products of WCSV-1 ORF IV and ORF VI from 10 bulb tissue samples of water chestnut using the primers of CP-F/R and RA-F/R, respectively. M: Marker II (TIANGEN Biotech, Beijing Co., Ltd.), Line 1-10: bulb samples; ck+: Small RNA sequencing water chestnut sample as positive control; ck2-:Taro sample. **C1** and **C2**: Direct PCR amplification of 543 and 875 bp products of WCSV-1 ORF IV and ORF VI from 10 root tissue samples of water chestnut using the primers of CP-F/R and RA-F/R, respectively. M: Marker II (TIANGEN Biotech, Beijing Co., Ltd.), Line 1-10: root samples; ck+: Small RNA sequencing water chestnut sample as positive control; ck2-: Taro sample.


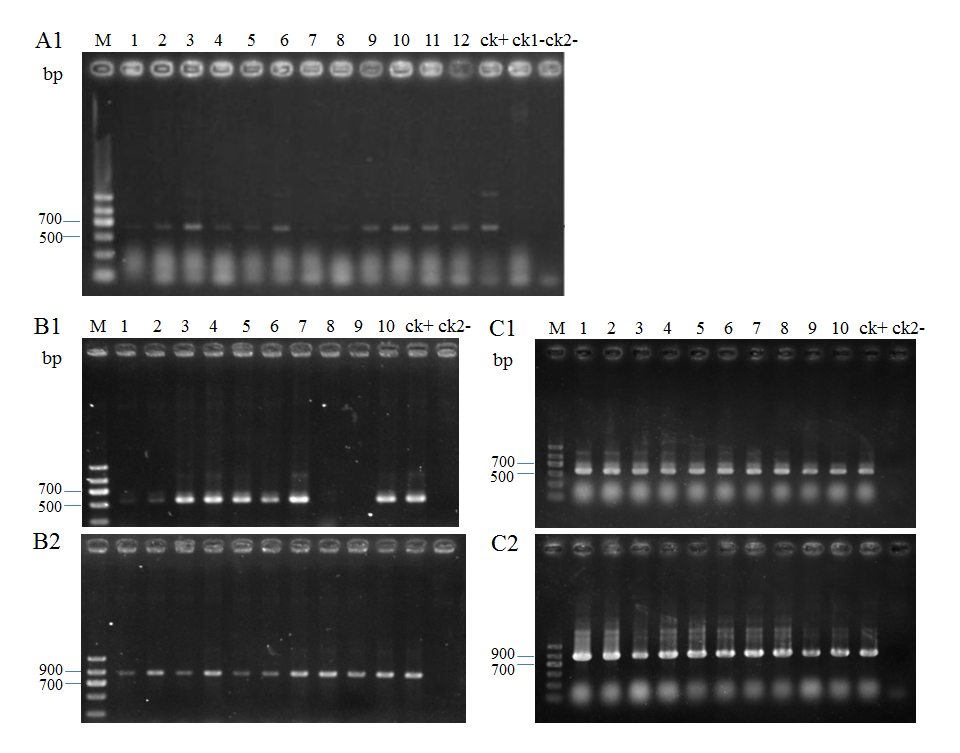

Supplement: Supplementary file 2 — Figure S2. 1.2% agarose gel electrophoresis of PCR products of WCSV-1 infecting water chestnut samples of “Tuanfeng” cultivar. A1: Direct PCR amplification of 650 bp products of WCSV-1 ORF I from 12 cladode samples of water chestnut using the primers of MP-F/R. M: Marker II (TIANGEN Biotech, Beijing Co., Ltd.), Line 1–12: Cladode tissue samples; ck+: Small RNA sequencing water chestnut sample as positive control; ck1-:ddH2O, ck2-: Taro sample. B1 and B2: Direct PCR amplification of 543 and 875 bp products of WCSV-1 ORF IV and ORF VI from 10 bulb tissue samples of water chestnut using the primers of CP-F/R and RA-F/R, respectively. M: Marker II (TIANGEN Biotech, Beijing Co., Ltd.), Line 1–10: bulb samples; ck+: Small RNA sequencing water chestnut sample as positive control; ck2-:Taro sample. C1 and C2: Direct PCR amplification of 543 and 875 bp products of WCSV-1 ORF IV and ORF VI from 10 root tissue samples of water chestnut using the primers of CP-F/R and RA-F/R, respectively. M: Marker II (TIANGEN Biotech, Beijing Co., Ltd.), Line 1–10: root samples; ck+: Small RNA sequencing water chestnut sample as positive control; ck2-: Taro sample. The samples were maintained at the greenhouse of the National Indoor Conservation Center for Virus-free Germplasm in Fruit Crops. (DOCX 330 kb) [file 12870_2019_1761_MOESM2_ESM.docx]
